# The supplementation of *Rothia* as a potential preventive approach for bone loss in mice with ovariectomy‐induced osteoporosis

**DOI:** 10.1002/fsn3.3747

**Published:** 2023-10-17

**Authors:** Ying‐Juan Li, Yuan‐Wei Zhang, Mu‐Min Cao, Ruo‐Lan Zhang, Meng‐Ting Wu, Yun‐Feng Rui, Nai‐Feng Liu

**Affiliations:** ^1^ Department of Geriatrics, Zhongda Hospital, School of Medicine Southeast University Nanjing Jiangsu PR China; ^2^ School of Medicine Southeast University Nanjing Jiangsu PR China; ^3^ Multidisciplinary Team (MDT) for Geriatric Hip Fracture Management, Zhongda Hospital, School of Medicine Southeast University Nanjing Jiangsu PR China; ^4^ Department of Orthopaedics, Zhongda Hospital, School of Medicine Southeast University Nanjing Jiangsu PR China; ^5^ Department of Cardiology, Zhongda Hospital, School of Medicine Southeast University Nanjing Jiangsu PR China

**Keywords:** bone loss, gut microbiota, osteoporosis, preventive approach, *Rothia*

## Abstract

There is an inseparable link between bone metabolism and gut microbiota, and the supplementation of probiotics exhibits a significant role in maintaining the homeostasis of gut microbiota and inhibiting bone loss. This study aims to explore the preventive and therapeutic potentials and the specific mechanisms of *Rothia* on osteoporosis. The mice models of osteoporosis induced by ovariectomy (OVX) were built, and the regular (once a day) and quantitative (200 μL/d) gavage of *Rothia* was performed for 8 weeks starting from 1 week after OVX. Microcomputed tomography was used to analyze the bone mass and bone microstructure of mice in each group after sacrifice. Histological staining and immunohistochemistry were then applied to identify the expression of pro‐inflammatory cytokines, intestinal permeability, and osteogenic and osteoclastic activities of mice. The collected feces of mice in each group were used for 16S rRNA high‐throughput sequencing to detect the alterations in composition, abundance, and diversity of gut microbiota. This study demonstrated that the gavage of *Rothia* alleviated bone loss in mice with OVX‐induced osteoporosis, improved OVX‐induced intestinal mucosal barrier injury, optimized intestinal permeability (zonula occludens protein 1 and occludin), reduced intestinal inflammation (tumor necrosis factor‐α and interleukin‐1β), and regulated imbalance of gut microbiota. Based on “gut‐bone” axis, this study revealed that regular and quantitative gavage of *Rothia* can relieve bone loss in mice with OVX‐induced osteoporosis by repairing the intestinal mucosal barrier injury, optimizing the intestinal permeability, inhibiting the release of pro‐inflammatory cytokines, and improving the disorder of gut microbiota.

## Graphical abstract

Based on “gut‐bone” axis, this study revealed that regular and quantitative gavage of *Rothia* can relieve the bone loss in mice with ovariectomy‐induced osteoporosis by repairing the intestinal mucosal barrier injury, optimizing the intestinal permeability, inhibiting the release of pro‐inflammatory cytokines, and improving the disorder of gut microbiota.

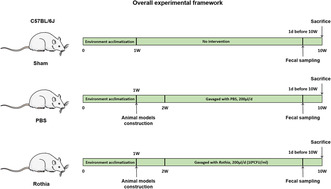

## 1 INTRODUCTION

With the increasingly severe aging process of the global population, the problem of osteoporosis faced by the elderly has become increasingly prominent (Zhang et al, 2022 ). Osteoporosis is a systemic and metabolic bone system disease characterized by low bone mass, destruction of bone microstructure, increased bone fragility, and enhanced fracture risk (Eastell & Szulc, 2017; Zhang, Cao, Li, Chen, Yu, & Rui, 2022; Zhang, Cao, Li, Zhang, Wu, Yu, & Rui, 2022). According to previous studies, more than 200 million people worldwide suffer from osteoporosis, and an osteoporotic fracture occurs every 3 s (Qiu et al., 2022; Tao et al., 2021). Hence, osteoporosis has become a major chronic disease that seriously influences the health of the elderly after cardiovascular disease and diabetes, known as the “silent killer,” which needs to attract sufficient attention from the whole society (Zhang, Cao, Li, Dai, Lu, Zhang, Bai, Chen, Zhang, et al., 2022; Zhang, Lu, Li, Dai, Cao, et al., 2021).

Gut microbiota refers to several symbiotic and pathogenic microbiotas residing on the surface of intestinal mucosa, including bacteria, archaea, viruses, fungi, and so on (Biver et al., 2019; Chevalier et al., 2020). Gut microbiota is also called as a “forgotten organ” because it contains more than 10^14^ types of bacteria and 150 times more genes than human body (Chen et al., 2017). In recent years, several studies have suggested that the gut microbiota of osteoporosis patients are different from that of healthy individuals, and the severity of bone loss in human body is also closely related to the changes in gut microbiota (Ding et al., 2022; Xie et al., 2022; Zhang, Li, Lu, Dai, Chen, & Rui, 2021). With the deepening of research and proposed concept of “gut‐bone” axis, the regulatory role and significance of gut microbiota in occurrence and development of osteoporosis have become a research hotspot recently (Zhang, Cao, Li, Lu, Dai, Zhang, Wang, & Rui, 2022). In addition, the supplementation of probiotics plays a crucial role in maintaining the homeostasis of gut microbiota and promoting the functional metabolism of multiple tissues and organs in whole body (Li et al., 2016; Zhang, Cao, Li, Dai, Lu, Zhang, Bai, Chen, Zhang, et al., 2022). Currently, several human and animal studies have reported that the supplementation of probiotics could promote the balance of osteoblast‐related bone formation and osteoclast‐related bone absorption via a variety of different mechanisms, and then inhibit bone loss and obtain the purpose of preventing and treating osteoporosis (Lambert et al., 2017; Rizzoli & Biver, 2020; Zhao et al., 2022).

*Rothia*, as one of the common components of gut microbiota, can secrete various molecules that interact with the host and other microbiota in digestive tract to improve intestinal biodiversity, enhance glucose tolerance, and help the colon cells recover vitality (Wei et al., 2020). Previous studies have indicated that the disorder of *Rothia* may contribute to metabolic and autoimmune diseases, such as irritable bowel syndrome, obesity, diabetes, asthma, allergies, fatty liver disease, rheumatoid arthritis, and systemic lupus erythematosus, by influencing multiple metabolic pathways (Fatima et al., 2021; Greve et al., 2021; Olshan et al., 2021; Sato et al., 2020). Meanwhile, as one of the members in the probiotic families, the supplementation of *Rothia* can reverse the course of diseases to a certain extent. Regarding this, Consolandi et al. (2015) compared the fecal microbiota of 22 patients with Behset's syndrome and 16 healthy cohabiting controls and observed that the number of *Rothia* in patients with Behset's syndrome was significantly reduced. Qian et al. (2020) analyzed the fecal microbiota of 40 patients with juvenile idiopathic arthritis and 42 healthy controls, and the results suggested that the relative abundance of *Rothia* in the feces of patients with juvenile idiopathic arthritis was significantly reduced. Seo et al. (2020) also conducted an experiment, and the results suggested that when the mice models of alcoholic fatty liver were supplemented with adequate *Rothia*, the condition of hepatic steatosis and inflammation was significantly improved, regardless of microbial activity. Therefore, it can be recognized that *Rothia* plays a crucial role in the construction of host homeostasis and the maintenance of body health. However, reports on the potential application of *Rothia* in the prevention and treatment of osteoporosis are still rare, and its effectiveness and safety also remain to be further explored. Based on this, this current study aims to explore the preventive and therapeutic potentials, and the specific mechanisms of *Rothia* on osteoporosis.

## 2 MATERIALS AND METHODS

### 2.1 Animal preparation and disposal

The animal experiment design schemes of this present study were approved by the institutional animal care and use committee of the School of Medicine, Southeast University (No. 20210510024). Female C57BL/6 mice aged 8 weeks were purchased from Gempharmatech Company (Nanjing, Jiangsu, China). The mice were raised under specific pathogen‐free conditions, the food and water were available ad libitum, the conditions of the breeding environment were maintained at 25 ± 2°C and 50% ± 5% humidity, and with a 12:12 h light/dark cycle.

### 2.2 Construction of ovariectomy (OVX)‐induced osteoporosis model

The experimental animal modeling was conducted after 1 week of environmental adaptation. Construction of OVX‐induced osteoporosis model was generally described previously (Sun et al., 2021); after being anesthetized with the 1% pentobarbital sodium solution (40 mg/kg), the mice were placed in a prone position and a longitudinal incision was made on the midline skin of the back (about 1 cm). The muscle fibers of each layer were then separated at the tip of tissue scissors, and the bilateral ovaries were located, identified, ligated, and resected in turn. Subsequently, the bleeding was stopped with the cotton swab, and the incision was sutured layer by layer with absorbable suture. Ultimately, the mice were placed on a warm blanket to wait for resuscitation.

### 2.3 Experimental grouping and sample collection

**FIGURE 1:**
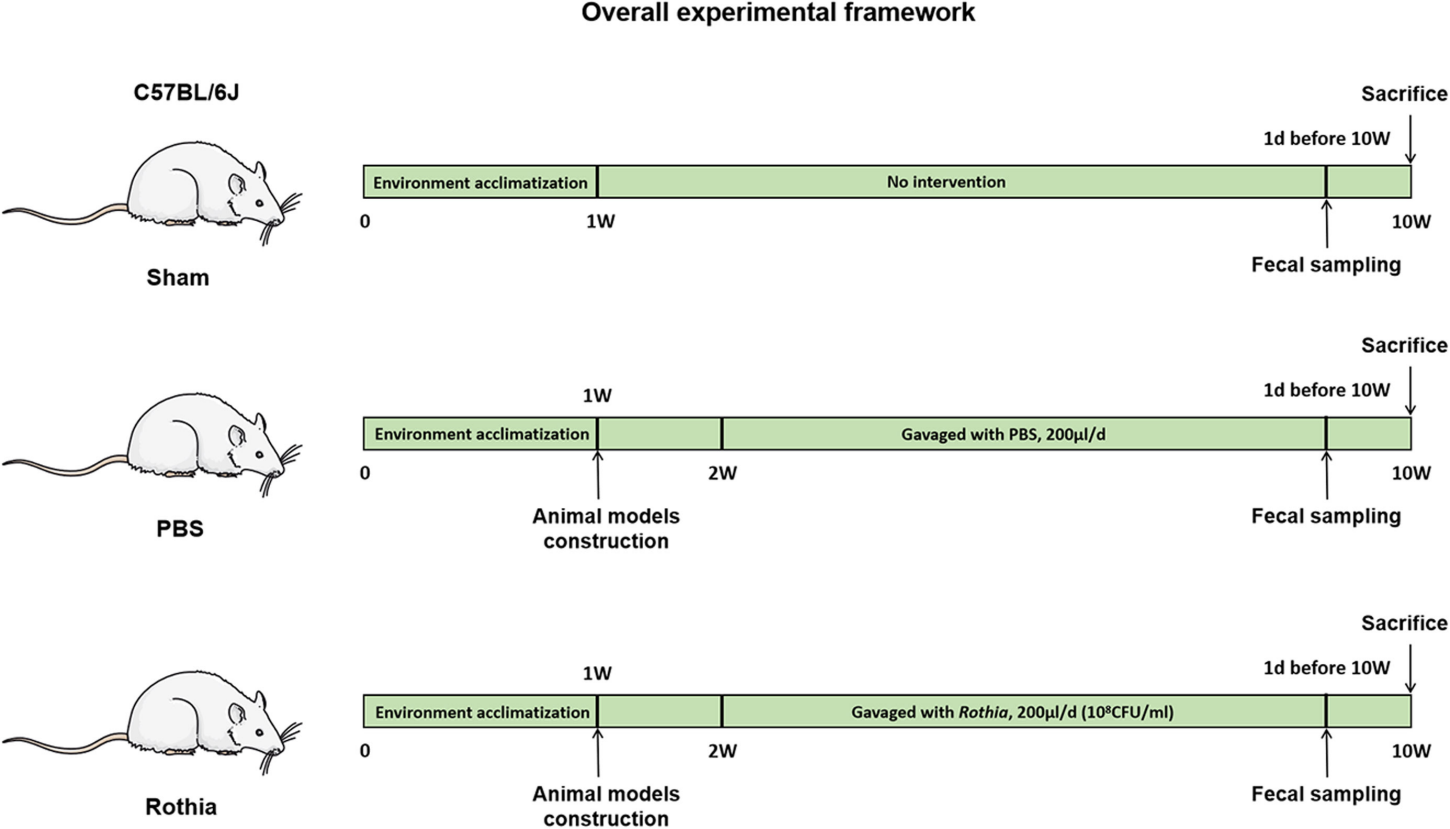
The overall experimental framework of this present study.

As exhibited in Figure 1, all included mice were divided into three groups, including the Sham group (*n* = 6), phosphate buffer solution (PBS) group (*n* = 6), and Rothia group (*n* = 6). Mice in the Sham group received the same procedures as above except that the ovaries were preserved. Based on the OVX, the mice in PBS group and Rothia group received PBS and *Rothia* by gavage once a day (200 μL) for continuous 8 weeks starting from 1 week after OVX. Therein, the level of supplementation of *Rothia* was mainly determined based on the previous research (Sun et al., 2018; Zhao et al., 2022). One day before the sacrifice, after the last gavage, the mice in each group were deprived of food and water for 12 h, and then the fresh feces of mice in each group were collected and immediately stored at −80°C. In the end, the mice were sacrificed and the femur and colon tissues of mice in each group were collected for further analysis.

### 2.4 Culture and preparation of *Rothia*

The *Rothia* strain freeze‐dried powder was purchased from the Guangdong Microbial Culture Collection Center (GDMCC). In this process, the lyophilized tube was taken out, and 0.5 mL of sterile water was sucked (balanced in an anaerobic environment for 24 h) and injected into the lyophilized tube. After the *Rothia* powder was fully dissolved, the solution was injected into two blood plates (200 μL/ piece) and evenly coated. Subsequently, the blood plates were placed in an anaerobic environment at 37°C and cultured for 24 to 48 h. After one to two generations (24 to 48 h) of culture, the *Rothia* strain (10^8^ CFU/mL) was revitalized (Uranga et al., 2020). After one to two generations of the *Rothia* strains were cultured, the culture medium containing the *Rothia* strains was transferred to centrifuge tube. Centrifugation was performed for 10 min at 4°C and 4000 rpm/min. After centrifugation, the supernatant was absorbed and filtered by a 0.22 μm filtration membrane two to three times to obtain the *Rothia* supernatant, which could be used in the gavage of mice in Rothia group.

### 2.5 Microcomputed tomography (micro‐CT) scanning and analysis

After the intervention was completed according to set period, mice in each group were sacrificed, and the bilateral femur tissues were collected for micro‐CT detection. Specifically, the femoral structures of mice were assessed by micro‐CT scanning device (SkyScan) with the specific scanning parameters (voltage: 70 kV, current: 200 μA, resolution: 18 μm). A 0.5‐mm‐thick aluminum filter was used to reduce the beam hardening in the process of micro‐CT scanning. Subsequently, the NRecon software (SkyScan) was used to reconstruct the image, and CTVol software (SkyScan) was used to visualize the 3D model. Then, a morphometric analysis was performed on 50 cross‐sections of the region of interest (ROI) under femoral growth plate, and a further analysis was performed on 200 cross‐sections of trabecular bone. Ultimately, CTAn program (SkyScan) was applied to analyze the bone tissue parameters of the femoral region, including bone mineral density (BMD), bone surface area/total volume (BS/TV), bone volume/total volume (BV/TV), bone surface/volume ratio (BS/BV), trabecular number (Tb.N), trabecular distance (Tb.Sp), trabecular thickness (Tb.Th), and structure model index (SMI).

### 2.6 Histological staining and analysis

After the completion of micro‐CT scanning, the femur and colon tissues of mice in each group were fixed in 4% paraformaldehyde for 24 h. Then, the femur tissue was decalcified with 12.5% ethylenediamine tetraacetic acid, and the decalcification cycle was 3 to 4 weeks. Subsequently, the decalcified femur tissue and the fixed colon tissue were further embedded in paraffin, and the 5‐μm‐thick tissue sections were prepared. Next, the hematoxylin–eosin (H&E) staining was performed on femur and colon tissue sections, and further tartrate‐resistant acid phosphatase (TRAP) staining was applied to evaluate the osteoclast generation in the femur tissue. In addition, the Image J software (National Institutes of Health, Bethesda) was used to quantitatively analyze the number of osteoclasts.

### 2.7 Immunohistochemistry (IHC) and analysis

The femur and colon sections of mice in each group were balanced in 0.1 M Tris‐buffered saline for 10 min. The sections were then placed in PBS and blocked with 10% normal goat serum for 1 h. Next, the femur sections were incubated overnight at 4°C with primary antibodies of the osteopontin (OPN), osteoprotegerin (OPG), recombinant runt‐related transcription factor 2 (RUNX2), receptor activator for nuclear factor‐κB ligand (RANKL), interleukin‐1β (IL‐1β), and tumor necrosis factor‐α (TNF‐α). Colon sections were incubated overnight at 4°C with primary antibodies of zonula occludens protein 1 (ZO‐1), occludin, IL‐1β, and TNF‐α. Subsequently, femur and colon sections were carefully washed in PBS for 15 min and incubated with secondary antibody bound with horseradish peroxidase at room temperature for 1 h. 3,3′‐diaminobenzidine was then used to visualize the expression of various specific markers under the microscope, and the Image J software (National Institutes of Health) was used to present positive cell regions under six randomly selected high‐power fields of view for quantitative analysis.

### 2.8 16S rRNA sequencing and bioinformatics analysis

On the day before mice were sacrificed, fresh feces from mice in each group were collected for the 16S rRNA high‐throughput sequencing. Specifically, 16S rRNA high‐throughput sequencing was based on Illumina Novase platform (Illumina), using double‐terminal sequencing method to construct a small fragment library for sequencing. The species composition of the samples could be revealed by splicing, filtering, clustering, or denoising of sequencing sequences, and the species annotation and abundance analysis (Johnson et al., 2019). The subsequent α‐diversity analysis, significant species difference analysis, correlation analysis, compositions and difference analysis, function prediction analysis, and so on can excavate the differences between samples (Abellan‐Schneyder et al., 2021). After the total DNA of the samples was extracted, primers were designed according to the conserved region, and sequencing connectors were added to the end of the primers for PCR amplification. Products were then purified, quantified, and homogenized to form sequencing libraries, the constructed libraries were inspected, and qualified libraries were sequenced. The original image data files obtained by high‐throughput sequencing were converted into original sequencing sequences by base identification and analysis, and the results were stored in the FASTQ file format, which contained the sequence information of reads and its corresponding sequencing quality information (Zheng et al., 2022).

### 2.9 Statistical assessment

Statistical assessment in this current study was conducted by SPSS 23.0 software (IBM), and the results were presented as the mean ± standard deviation. A *t*‐test was used to compare the data between two groups, and one‐way ANOVA test was used to compare the data between multiple groups. A *p* < .05 was considered statistically significant, and each experiment included at least three repeated results.

## 3 RESULTS

### 3.1 The gavage of *Rothia* alleviated bone loss in mice with OVX‐induced osteoporosis

**FIGURE 2:**
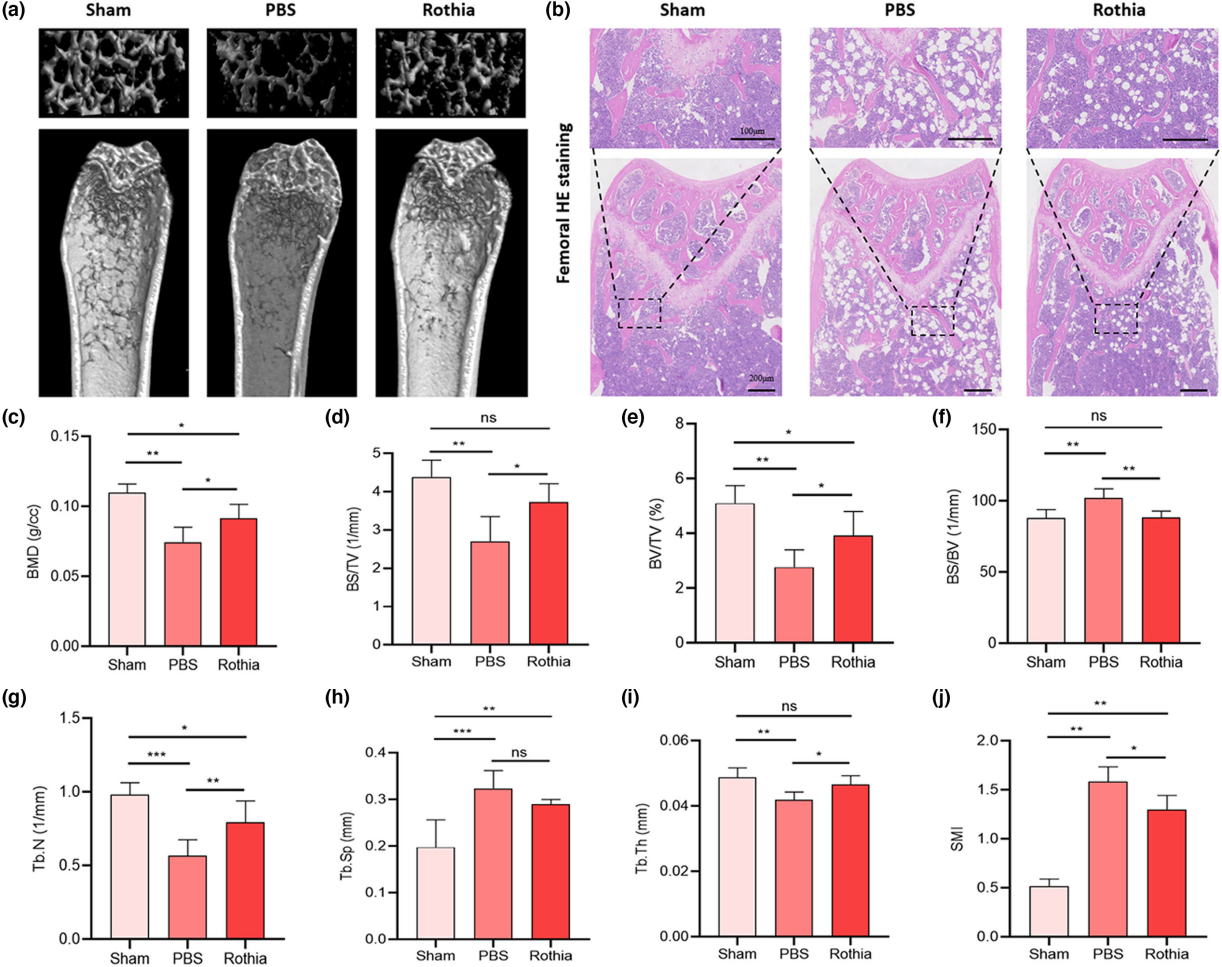
The gavage of *Rothia* alleviated bone loss in mice with OVX‐induced osteoporosis. (a) Micro‐CT scanning images of ROI region of distal femur of mice in each group; (b) Femoral H&E staining of mice in each group. Scale bars represented as 100 and 200 μm; (c) BMD; (d) BS/TV; (e) BV/TV; (f) BS/BV; (g) Tb.N; (h) Tb.Sp; (i) Tb.Th; and (j) SMI on the femur trabecular bone were analyzed by micro‐CT. *p < .05, **p < .01, and ***p < .001 compared with comparable group. BMD, bone mineral density; BS/BV, bone surface/volume ratio; BS/TV, bone surface area/total volume; BV/TV, bone volume/total volume; H&E, hematoxylin–eosin; micro‐CT, microcomputed tomography; OVX, ovariectomy; ROI, region of interest; SMI, structure model index; Tb.N, trabecular number; Tb.Sp, trabecular distance; Tb.Th, trabecular thickness.

**FIGURE 3:**
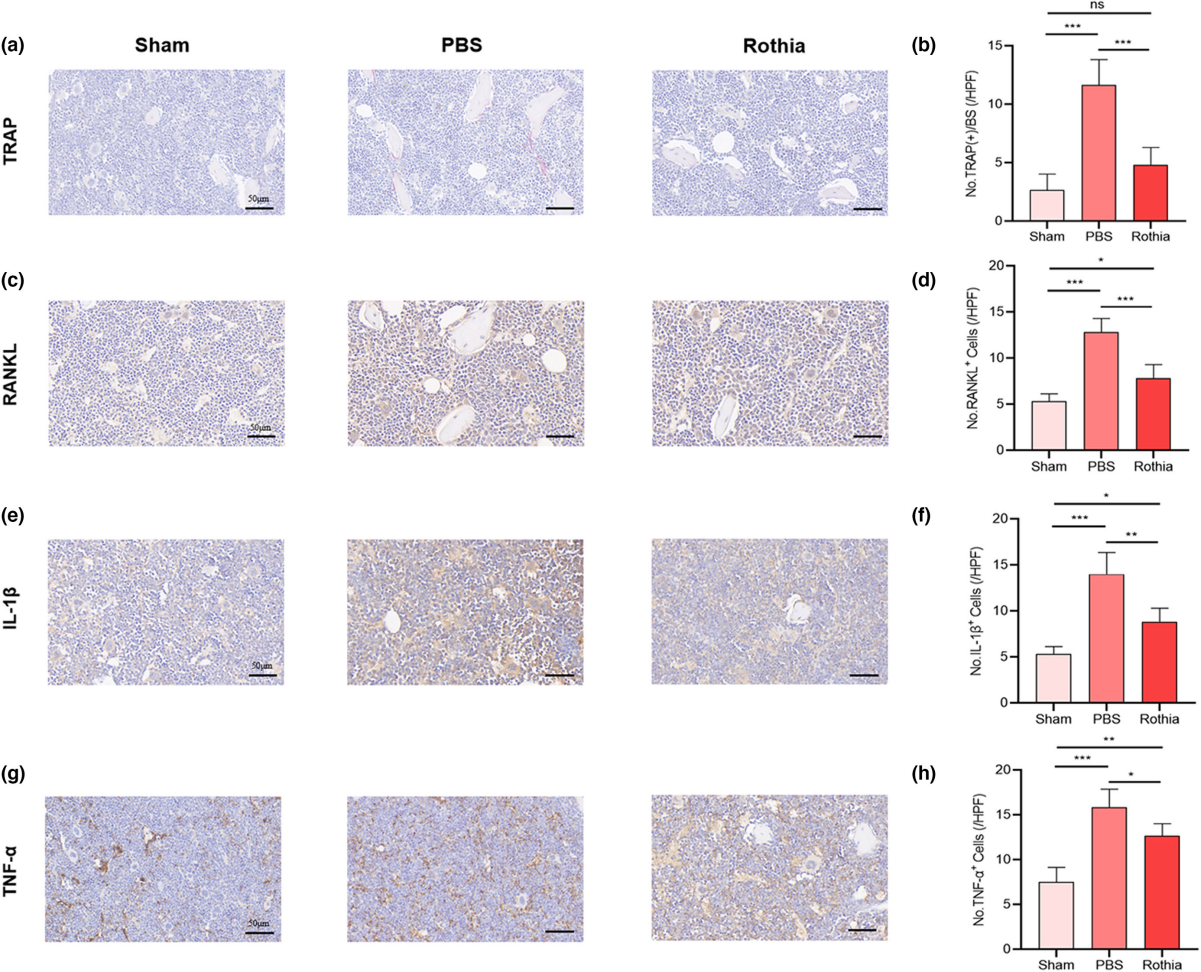
The gavage of *Rothia* inhibited osteoclastogenesis. (a) TRAP stained the sections of femur tissues. Scale bars represented as 50 μm; (b) No. TRAP^+^ cells; (c) RANKL stained the sections of femur tissues. Scale bars represented as 50 μm; (d) No. RANKL^+^ cells; (e) IL‐1β stained the sections of femur tissues. Scale bars represented as 50 μm; (f) No. IL‐1β^+^ cells; (g) TNF‐α stained the sections of femur tissues. Scale bars represented as 50 μm; (h) No. TNF‐α^+^ cells. *p < .05, **p < .01, and ***p < .001 compared with comparable group. IL‐1β, interleukin‐1β; RANKL, receptor activator for nuclear factor‐κB ligand; TRAp, tartrate‐resistant acid phosphatase; TNF‐α, tumor necrosis factor‐α.

After the completion of experimental cycle, we detected the distal femoral structure of mice in each group via micro‐CT scanning, and selected 50 cross‐sections (0.5 mm) under the distal femoral growth plate as ROI regions for quantitative morphometric analysis. The results suggested that, compared with the morphology of distal femur in Sham group, the cortical bone of distal femur in PBS group was thinner, the cancellous bone was sparser, and the number of bone trabeculae was reduced accordingly. Through *Rothia* gavage once a day for 8 consecutive weeks, the loss of cancellous bone in mice in Rothia group was improved, and the number of bone trabeculae was retained (Figure 2a). This result was also confirmed by the results of femoral H&E staining (Figure 2b) and TRAP staining (Figure 3a,b). Based on the scanning images, we analyzed various imaging parameters related to micro‐CT (Figure 2c–j). The results suggested that BMD (*p* < .05), BS/TV (*p* < .05), BV/TV (*p* < .05), Tb.N (*p* < .01), and Tb.Th (*p* < .05) in the ROI region of distal femur of mice in Rothia group were enhanced compared with those in the PBS group, while the values of BS/BV (*p* < .01) and SMI (*p* < .05) were reduced. There was no significant difference in the value of Tb.Sp (*p* > .05).

**FIGURE 4:**
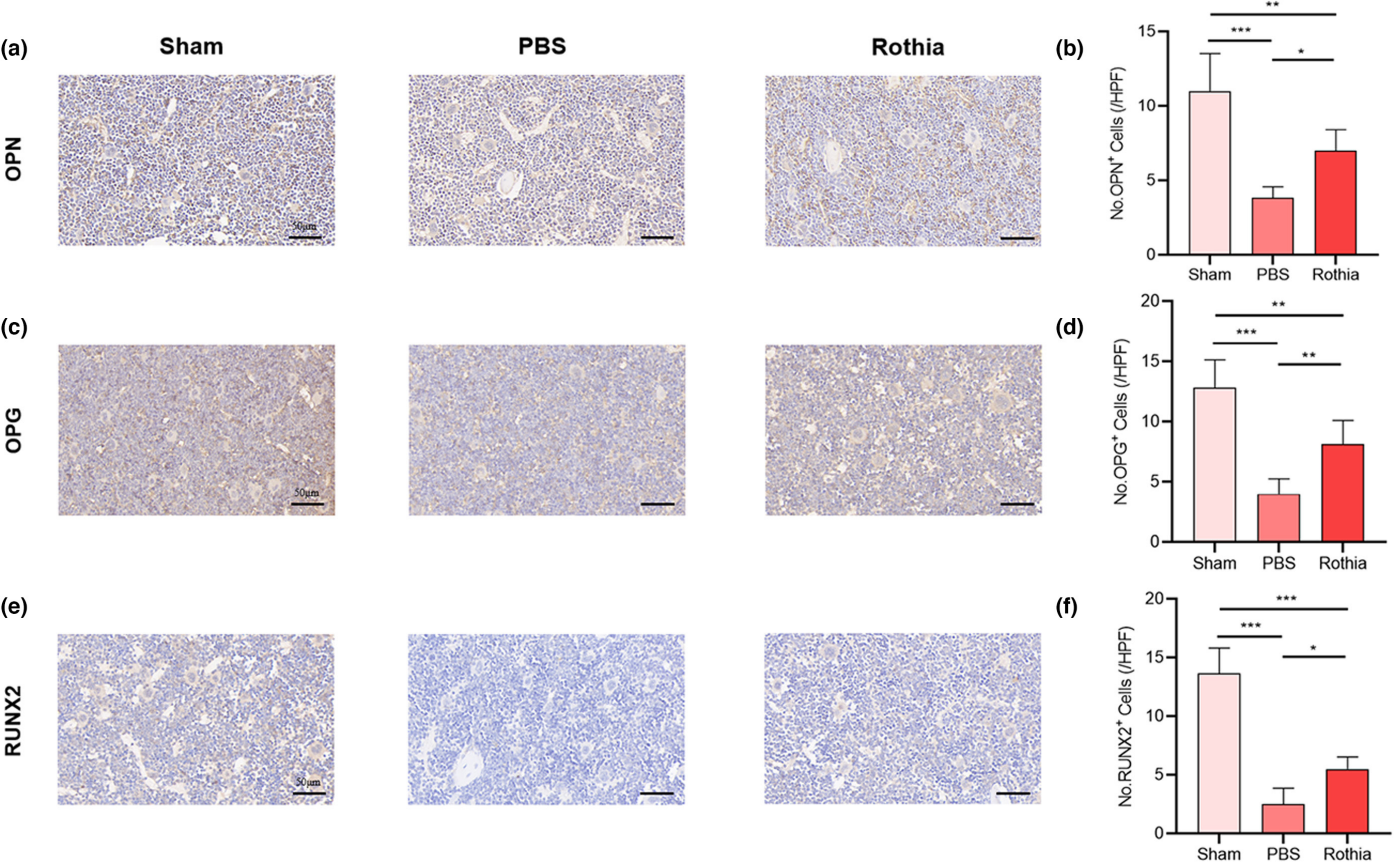
The gavage of *Rothia* promoted the osteogenesis. (a) OPN stained sections of femur tissues. Scale bars represented as 50 μm; (b) No. OPN^+^ cells; (c) OPG stained the sections of femur tissues. Scale bars represented as 50 μm; (d) No. OPG^+^ cells; (e) RUNX2 stained sections of femur tissues. Scale bars represented as 50 μm; (f) No. RUNX2^+^ cells. *p < .05, **p < .01, and ***p < .001 compared with comparable group. OPG, osteoprotegerin; OPN, osteopontin; RUNX2, recombinant runt‐related transcription factor 2.

As for IHC analysis, the results indicated that the expression of RANKL enhanced after OVX, and the regular and quantitative gavage of *Rothia* decreased the expression of RANKL relative to that in PBS group (Figure 3c,d). Meanwhile, the expression of IL‐1β and TNF‐α was also measured in femur tissue, and the results showed the enhanced expression of IL‐1β and TNF‐α after OVX, whereas the regular and quantitative gavage of *Rothia* decreased the expression of IL‐1β and TNF‐α in the femur tissue (Figure 3e–h). Moreover, the results also indicated that the expressions of OPN, OPG, and RUNX2 reduced after OVX, and the regular and quantitative gavage of *Rothia* increased the expression of OPN, OPG, and RUNX2 relative to that in PBS group (Figure 4a,f). In combination with above results, the visual evidence of *Rothia*'s role in alleviating OVX‐induced osteoporosis can be observed, which may be closely involved in the bone regulation by reducing the levels of pro‐inflammatory cytokines and then obtaining the balance between bone resorption and bone formation.

### 3.2 The gavage of *Rothia* improved OVX‐induced intestinal mucosal barrier injury

**FIGURE 5:**
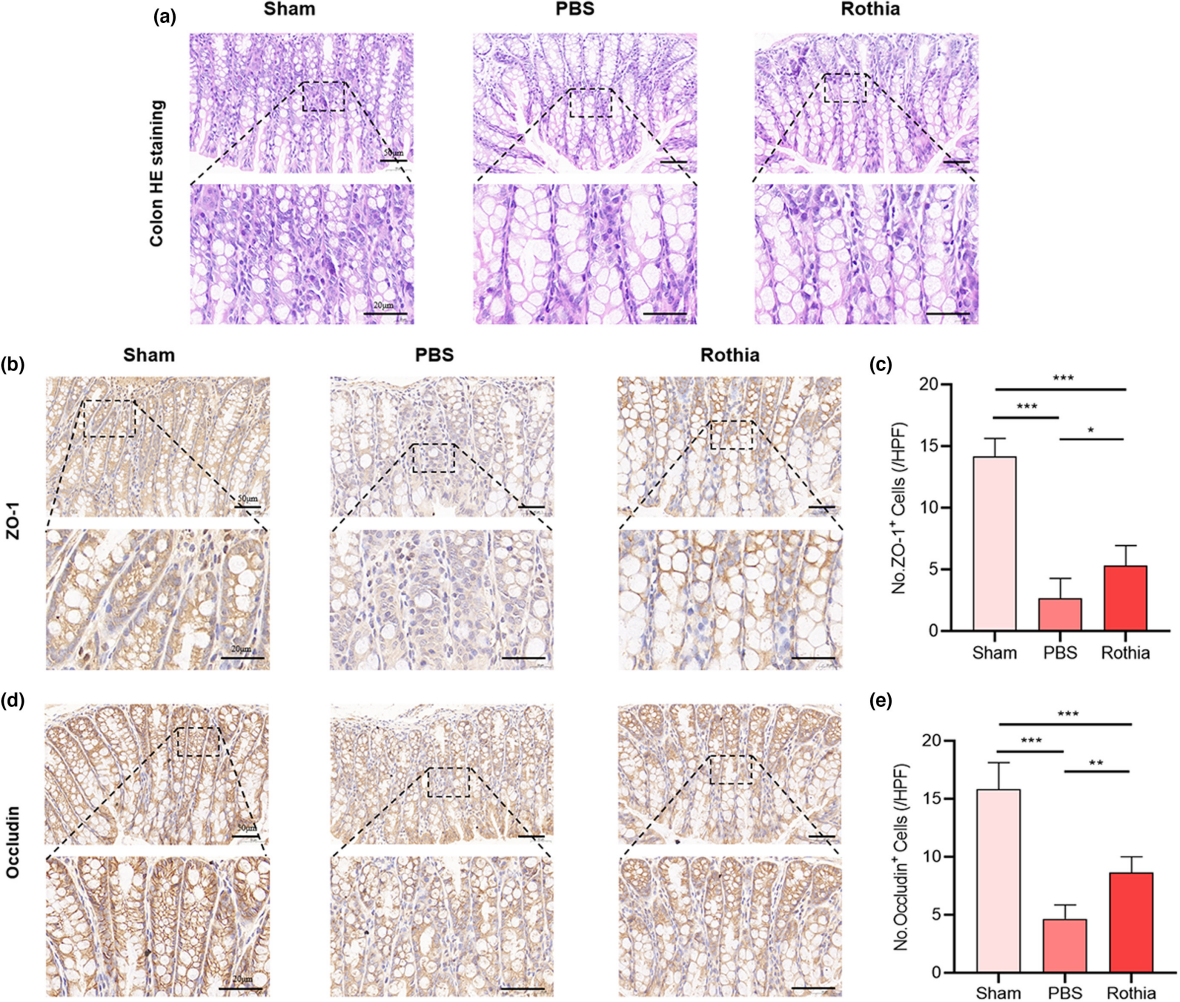
The gavage of *Rothia* improved the OVX‐induced intestinal mucosal barrier injury. (a) H&E staining of colon tissues. Scale bars represented as 50 and 20 μm; (b) ZO‐1 stained the sections of colon tissues. Scale bars represented as 50 μm and 20 μm; (c) No. ZO‐1^+^ cells; (d) Occludin stained the sections of colon tissues. Scale bars represented as 50 and 20 μm; (e) No. Occludin^+^ cells. *p < .05, **p < .01, and ***p < .001 compared with comparable group. H&E, hematoxylin–eosin; OVX, ovariectomy; ZO‐1, zonula occludens protein 1.

In terms of the density of intestinal mucosal barrier, the results of H&E staining of colon tissue of the mice suggested that the intestinal cavity of mice in PBS group was sparser than that in Sham group, and the intestinal space was significantly enlarged. However, the regular and quantitative gavage of *Rothia* improved the intestinal cavity density of mice in Rothia group, and the intestinal space was optimized correspondingly, indicating a level comparable to that of mice in the Sham group (Figure 5a). IHC results (Figure 5b–e) revealed that the expression of tight junction component proteins (including occludin and ZO‐1) in the intestine of mice in PBS group decreased (*p* < .001), while the regular and quantitative gavage of *Rothia* improved that in Rothia group (*p* < .05), suggesting that the gavage of *Rothia* could contribute to repairing the injured intestinal mucosal barrier in mice with OVX‐induced osteoporosis and optimizing the intestinal permeability.

### 3.3 The gavage of *Rothia* reduced intestinal inflammation induced by OVX

**FIGURE 6:**
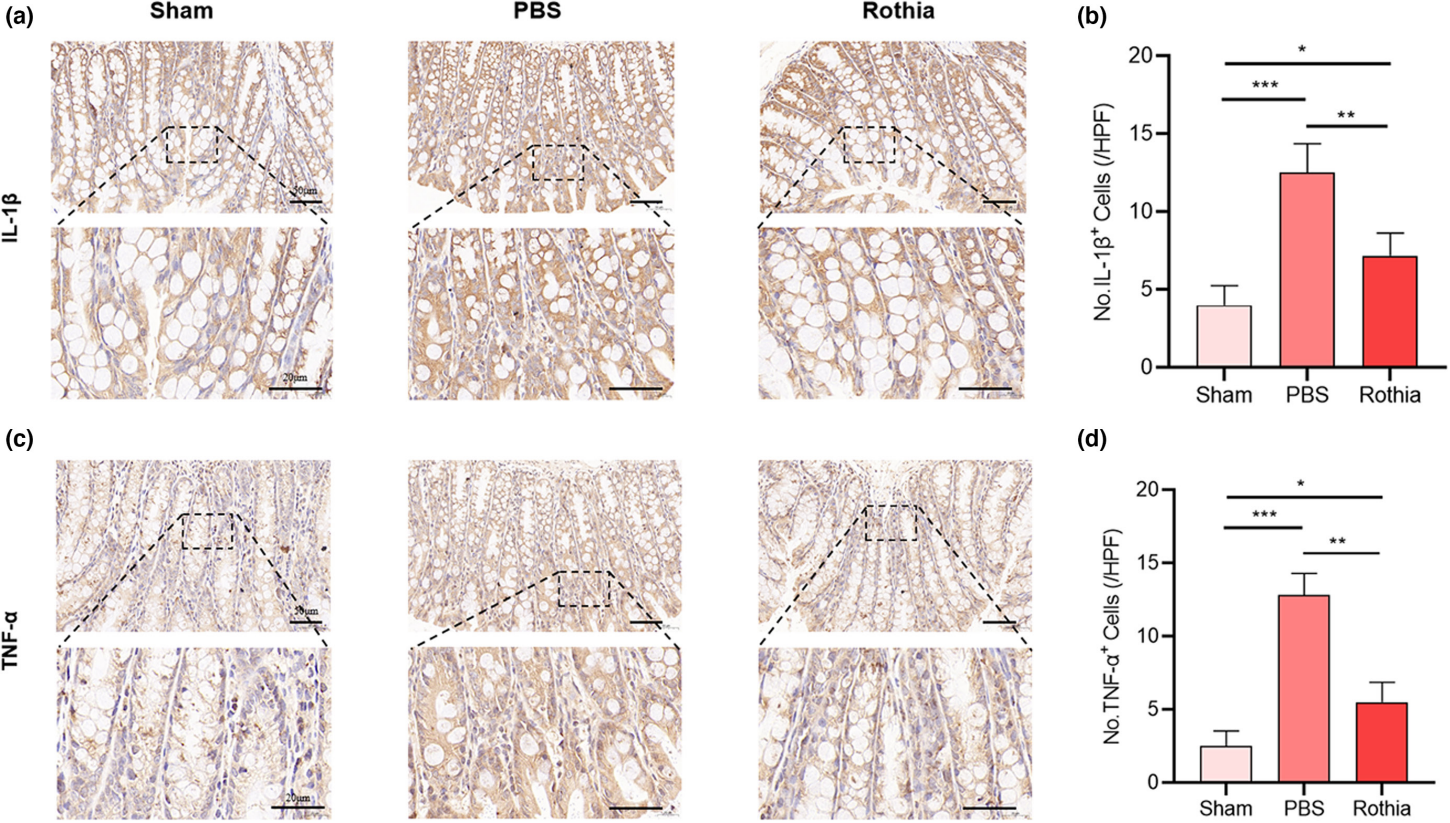
The gavage of *Rothia* reduced intestinal inflammation induced by OVX. (a) IL‐1β‐stained sections of colon tissues. Scale bars represented as 50 and 20 μm; (b) No. IL‐1β^+^ cells; (c) TNF‐α‐stained sections of colon tissues. Scale bars represented as 50 and 20 μm; (d) No. TNF‐α^+^ cells. *p < .05, **p < .01, and ***p < .001 compared with comparable group. IL‐1β, interleukin‐1β; OVX, ovariectomy; TNF‐α, tumor necrosis factor‐α.

Accordingly, with the enhancement of intestinal permeability, the IHC results in Figure 6 also showed that the expression of intestinal inflammatory indicators (including IL‐1β and TNF‐α) of the mice in PBS group was significantly higher than that in Sham group (*p* < .001). However, after the regular and quantitative gavage of *Rothia*, the expression of intestinal inflammatory indicators of mice in Rothia group was decreased (*p* < .01). Based on the above results, it can be recognized that regular and quantitative gavage of *Rothia* reduced intestinal permeability and improved intestinal inflammation by repairing the OVX‐induced intestinal mucosal barrier function in mice.

### 3.4 The gavage of *Rothia* regulated imbalance of gut microbiota in mice with OVX‐induced osteoporosis

**FIGURE 7:**
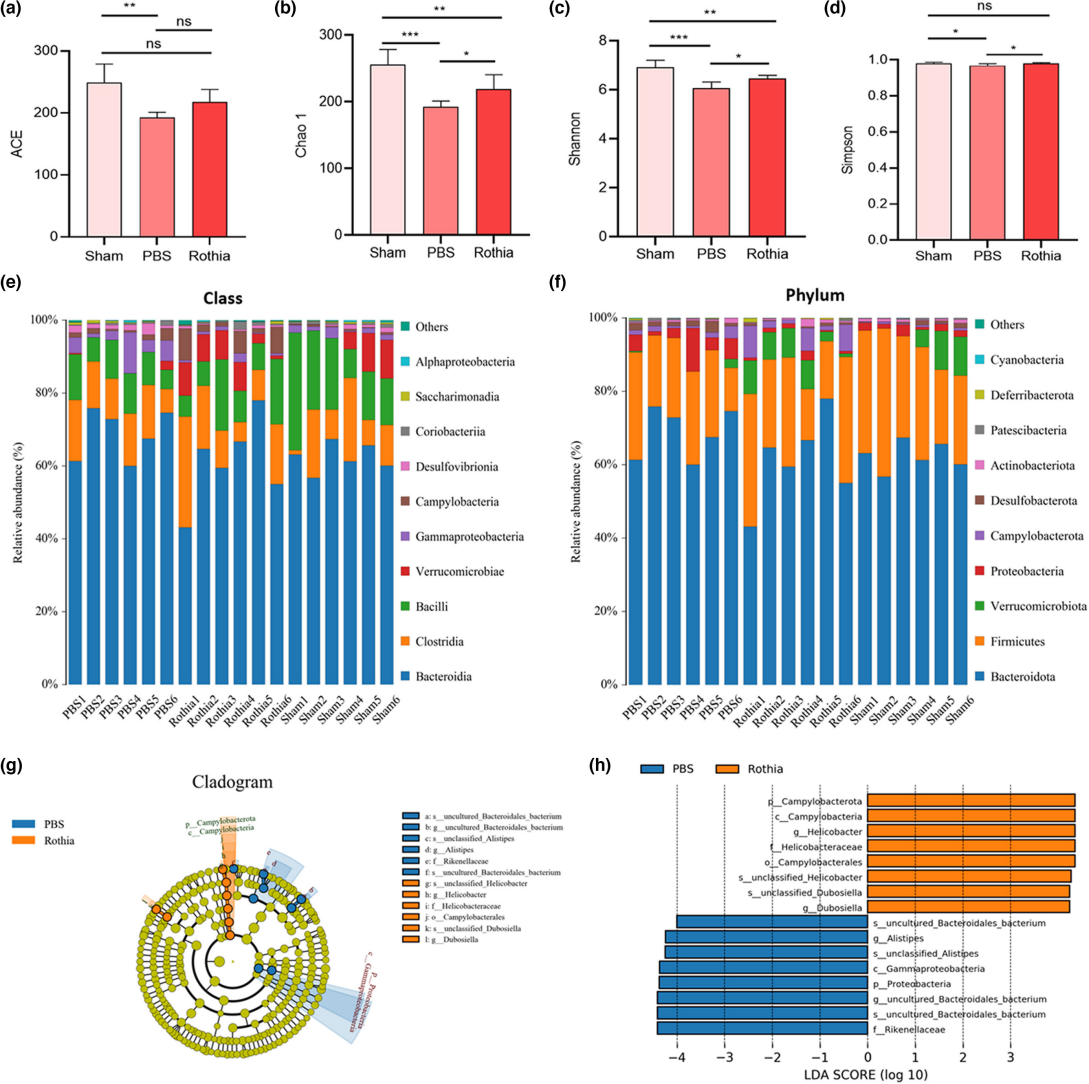
The gavage of *Rothia* regulated the imbalance of gut microbiota in mice with OVX‐induced osteoporosis. (a) ACE index; (b) Chao 1 index; (c) Shannon index; (d) Simpson index; (e) composition of gut microbiota at class level; (f) Composition of gut microbiota at phylum level; (g) the cladogram showing the difference in microbial taxonomic composition in different groups; (h) LEfSe analysis for the identification of the microbial taxonomic markers. *p < .05, **p < .01, and ***p < .001 compared with comparable group. OVX, ovariectomy.

One day before the sacrifice of mice in each group and after the completion of last gavage, the mice in each group were fasted for 12 h, and then the feces were collected and stored at −80°C. Next, the 16S rRNA high‐throughput sequencing was conducted. The results showed that there were differences in ACE, Chao1, Shannon, and Simpson indexes between PBS group and Sham group in terms of α‐diversity (*p* < .05), and the regular and quantitative gavage of *Rothia* helped restore the abundance and diversity of microbiota of mice in Rothia group. Specifically, in terms of the Chao1, Shannon, and Simpson indexes, the mice in Rothia group exhibited higher microbiota abundance and diversity than that in PBS group (*p* < .05, Figure 7a–d). Besides, in terms of the structure and composition of microbiota, the regular and quantitative gavage of *Rothia* was also beneficial to the recovery of gut microbiota at various taxonomic levels in mice with OVX‐mediated osteoporosis (Figure 7e,f). In addition, the cladogram was generated and significant differences in microbial taxonomic composition occurred in different groups (Figure 7g), and the LEfSe analysis was further performed to identify microbial taxonomic markers (Figure 7h).

### 3.5 The function prediction analysis of gut microbiota

**FIGURE 8:**
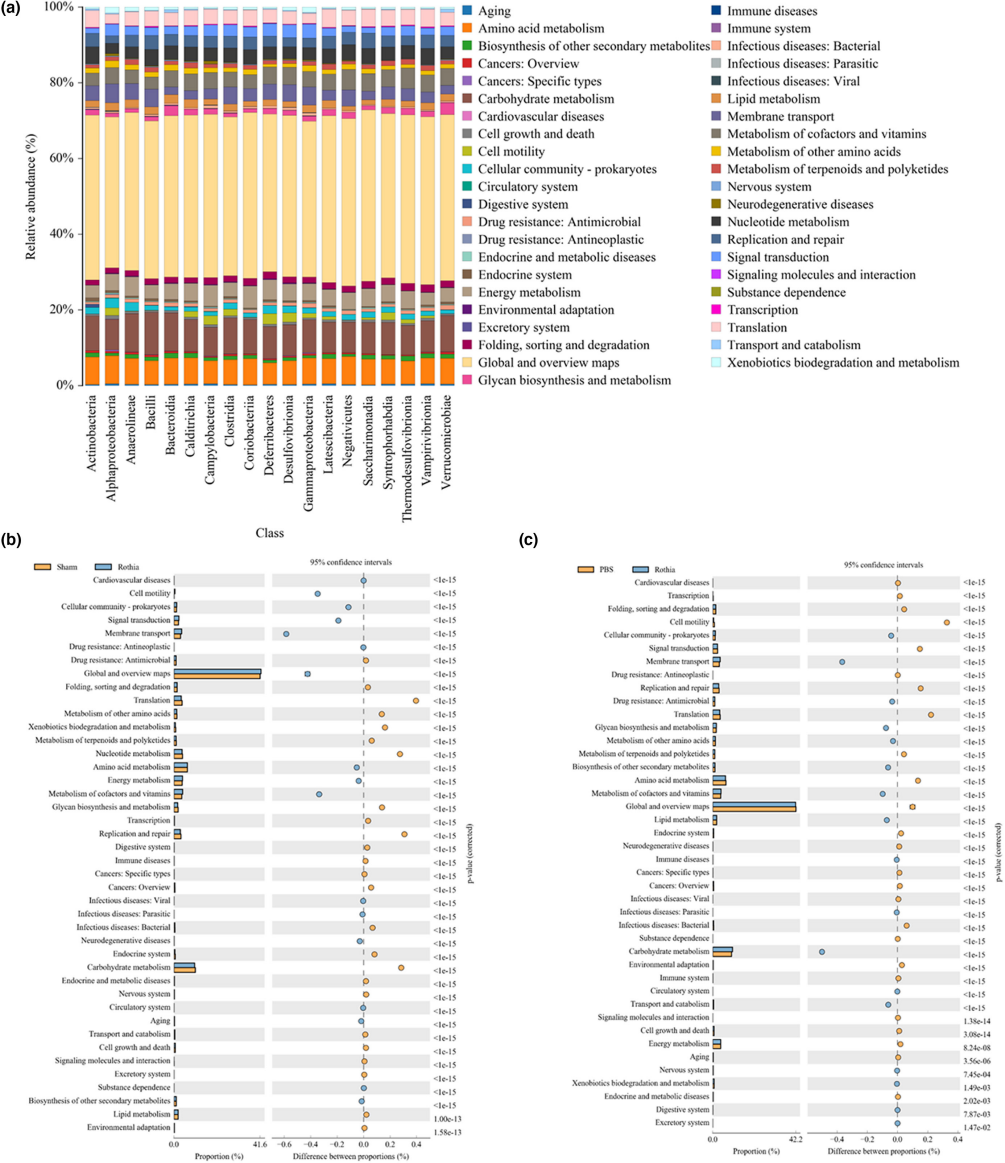
Function prediction analysis of gut microbiota. (a) Functional contribution of microbiota predicted by compositions and difference analysis; (b) and (c) comparisons of the functional contribution of microbiota between *Rothia* group and other two groups.

Based on the alterations of gut microbiota among these groups, the function prediction analysis of gut microbiota was further conducted, and the functional contents of metagenome were also inferred. As exhibited in Figure 8a, the functional contribution of microbiota was predicted via the means of compositions and difference analysis, and the comparisons between Rothia group and other two groups are shown in Figure 8b,c. In detail, the terms of cell motility, signal transduction, membrane transport, amino acid metabolism, translation, replication, and repair, global and overview maps, lipid metabolism, and transport and catabolism in Rothia group were significantly different from those of PBS group (Figure 8c).

## 4 DISCUSSION

With the continuous advancement of the aging process of global population, the number of middle‐aged and elderly people around the world has increased dramatically (Zhang, Cao, Li, Chen, Yu, & Rui, 2022; Zhang, Lu, Li, Dai, Chen, et al., 2021), causing an increasingly heavy social burden caused by osteoporosis and osteoporosis‐related fragile fractures (Watts et al., 2021; Zhang, Lu, Li, Wang, Zhao, Chen, & Rui, 2022). Meanwhile, several previous studies have reported the disorder of gut microbiota in patients with osteoporosis and OVX‐induced osteoporosis mice models, indicating that there is an inseparable relationship between gut microbiota and bone metabolism (Li et al., 2021; Liu et al., 2021). In this context, more and more researches in recent years suggested that the supplementation of probiotics could play a crucial role in maintaining the homeostasis of gut microbiota and promoting the functional metabolism of multiple organs and systems of the body (Hernandez et al., 2016; Torres et al., 2019). In this present study, we demonstrated that the regular and quantitative gavage of *Rothia* can alleviate bone loss in mice with OVX‐induced osteoporosis by repairing the intestinal mucosal barrier injury, optimizing the intestinal permeability, inhibiting the release of intestinal pro‐inflammatory cytokines, and improving the disorder of gut microbiota. Therein, as for the transmission of pro‐inflammatory cytokines, the blood circulation may act as a bridge in the bidirectional relationship of the “gut‐bone” axis, and further becomes the intermediate medium of communication and interaction between the gut and bone.

The gut microbiota in intestinal tract is mainly composed of beneficial microbiota and harmful microbiota, which correspondingly play two opposite influences on bone metabolism (Shi et al., 2022). Probiotics mainly stimulate the activities of carbohydrate microbiota, increase the secretion of organic acids, cause the decrease in intestinal PH (contributing to building an acidic microenvironment), enhance the intestinal antibacterial ability, thus inhibiting the growth of harmful microbiota, and further maintain the balance of gut microbiota and the stability of intestinal function (Tu et al., 2021). In recent years, several human and animal researches have reported that the supplementation of probiotics can promote osteoblast‐related bone formation and osteoclast‐related bone absorption to obtain balance via a variety of different mechanisms, and then inhibit bone loss and achieve the purpose of preventing and treating osteoporosis.

On the level of human research, Li, Ji, et al. (2022) revealed in a randomized controlled trial that supplementing probiotics (*Lactobacillus reuteri ATCC PTA 6475*) with a cycle of 12 months could be beneficial to correct the disorder of gut microbiota of elderly women with low BMD and reverse the deterioration of their intestinal inflammation, thereby having beneficial influences on bone metabolism. Takimoto et al. (2018) included 76 healthy postmenopausal women in a randomized, placebo‐controlled, double‐blind clinical trial and treated with placebo or probiotics (*Bacillus subtilis C‐3102*) for 24 weeks, and the results indicated that compared with placebo, the supplementation of probiotics could improve the state of low BMD by inhibiting the bone absorption and regulating the disorder of gut microbiota. Jansson et al. (2019) also observed through a randomized, double‐blind, placebo‐controlled, multicenter trial that the lumbar BMD of postmenopausal women was improved after receiving the combination of probiotic strains (*Lactobacillus paracasei DSM13434*, *Lactobacillus plantarum DSM 15312*, and *Lactobacillus plantarum DSM 15313*) for 12 months, indicating that the combination of three probiotic strains can prevent bone loss in postmenopausal women.

In terms of animal research, Britton et al. (2014) conducted a 4‐week gavage of *Lactobacillus reuteri ATCC PTA 6475* in mice with OVX‐induced osteoporosis, and observed that the supplementation of probiotics inhibited the OVX‐induced increase in bone marrow CD4+T lymphocytes (promoting generation of osteoclasts), and directly inhibited the formation of osteoclasts in vitro, thus correcting the bone loss associated with estrogen deficiency, and was considered to be a simple and cost‐effective approach to reduce the bone loss after estrogen deficiency. Yuan and Shen (2021) showed in a previous experiment that the supplementation of probiotics (*Bacteroides vulgatus ATCC8482*) in mice with OVX‐induced osteoporosis for 8 consecutive weeks could correct the disorder of gut microbiota, inhibit the LPS/TLR‐4/p‐NF‐κB pathway in colon, reduce the level of TNF‐α in serum, and then inhibit the bone loss and the destruction of bone microstructure. Dar et al. (2018) also revealed via an experiment that the administration of probiotics (*Bacillus clausii*) to the mice with OVX‐induced osteoporosis for 6 weeks could restore the balance of Treg‐Th17 cells (increasing Treg cells and decreasing Th17 cells), and inhibit the expression of pro‐inflammatory cytokines (IL‐6, IL‐17, and TNF‐ α) and enhance the expression of anti‐inflammatory cytokines (IL‐10), so as to reduce the bone loss caused by estrogen deficiency. Regarding this, several previous studies have also reported that the supplementation of different types of probiotics is able to promote the expression of anti‐inflammatory cytokines and reduce expression of pro‐inflammatory cytokines, thus alleviating the intestinal and systemic inflammation and promoting the expression of immunoglobulin A to enhance the immune system of body (Li, Zhang, et al., 2022; Liu et al., 2022). More importantly, the supplementation of probiotics can also directly repair damaged intestinal mucosal barrier, inhibit intestinal inflammation, and thus preventing the pro‐inflammatory cytokines from acting on the bones of body with the blood circulation (Zhang, Cao, Li, Lu, Dai, Zhang, Wang, & Rui, 2022; Zhang, Cao, Li, Zhang, Wu, Yu, & Rui, 2022; Zhou et al., 2023). Liu et al. (2019) showed via a study that the administration of *Lactobacillus rhamnosus GG* for 8 consecutive weeks can reverse the bone loss induced by tenofovir fumarate diester in mice by repairing the integrity of intestinal mucosal barrier, increasing Treg cells and reducing Th17 cells, and downregulating the osteoclast‐related factors in bone marrow, spleen, and intestine. Thus, it is recognized that probiotic supplementation could improve bone metabolism by regulating the gut microbiota, and the anti‐osteoporosis potentials of partial probiotic strains have also been verified in several different experimental animal models related to osteoporosis.

It is worth mentioning that the current results are also similar to the recent study reported by Wang et al. (2021). In their study, the fecal samples of postmenopausal women were first detected by 16S rRNA high‐throughput sequencing, and *Prevotella histicola* was verified to be the key differential microbiota among different groups. Then, OVX‐induced osteoporosis models were further constructed and subjected to the gavage of *Prevotella histicola*, and the results suggested that *Prevotella histicola* has a protective effect on the bone mass, and the related mechanisms were related to the improvement of intestinal permeability, the correction of disorder of gut microbiota, and the reduction in release of pro‐inflammatory cytokines. Hence, the protective effects of various kinds of probiotics on bone mass have been supported by certain research evidence, but further exploration is still needed in the future to clarify its deeper mechanisms.

Ultimately, certain shortcomings and potential improvements in this present study still need to be pointed out. First, at the current stage, the 16S rRNA high‐throughput sequencing technology is still difficult to identify whether the microbiota is active or not and lacks the resolution capability above the genus level (Van Reckem et al., 2020), while this point could be solved with the improvement and progression of sequencing means in the near future. Second, the sample size of this present study is relatively small, and it is still necessary to conduct animal experimental verification and human prospective clinical trials with a larger sample size in the future. Third, the transformation of probiotics from animal studies to clinical practice is still faced with various challenges, including determination of safety, effectiveness, dosage, duration, and combination mode of probiotic products, and more randomized controlled trials with higher levels of evidence are still needed for verification in the future (Zhou et al., 2023). Nevertheless, we also have reasons to believe that with further promotion of subsequent studies and continuous innovation and development of research techniques, gut microbiota may become a significant target for regulating bone metabolism, and further regulation of gut microbiota through probiotics to prevent or treat osteoporosis is expected to become a promising intervention approach.

## 5 CONCLUSION

To sum up, based on the “gut‐bone” axis, this present study revealed that the regular and quantitative gavage of *Rothia* can relieve bone loss in mice with OVX‐induced osteoporosis by repairing the intestinal mucosal barrier injury, optimizing the intestinal permeability, inhibiting the release of pro‐inflammatory cytokines, and improving the disorder of gut microbiota. The regulation of gut microbiota via *Rothia* is expected to become a promising preventive approach for bone loss, although it is still necessary to conduct animal experimental verification and human prospective clinical trials with a larger sample size in the future.

## AUTHOR CONTRIBUTIONS

**Ying‐Juan Li:** Conceptualization (equal); data curation (equal); formal analysis (equal); funding acquisition (equal). **Yuan‐Wei Zhang:** Data curation (equal); formal analysis (equal); investigation (equal). **Mu‐Min Cao:** Data curation (equal); formal analysis (equal). **Ruo‐Lan Zhang:** Data curation (equal); formal analysis (equal); investigation (equal). **Meng‐Ting Wu:** Data curation (equal); methodology (equal). **Yun‐Feng Rui:** Data curation (equal); formal analysis (equal); funding acquisition (equal); investigation (equal). **Nai‐Feng Liu:** Conceptualization (equal); data curation (equal); formal analysis (equal); investigation (equal).

## CONFLICT OF INTEREST STATEMENT

The authors declare that they have no conflict of interest.

## ETHICS STATEMENT

The animal experiment design schemes of this present study were approved by the institutional animal care and use committee (IACUC) of the School of Medicine, Southeast University (No. 20210510024).

## INFORMED CONSENT

Not applicable.

## Data Availability

This is an open‐access article under the terms of the Creative Commons Attribution License, which permits use, distribution, and reproduction in any medium, provided the original work is properly cited.
